# Provider payment to primary care physicians in China: background, challenges, and a reform framework

**DOI:** 10.1017/S146342361800021X

**Published:** 2018-04-05

**Authors:** Xiaoying Pu, Yaming Gu, Xiaohe Wang

**Affiliations:** 1 Professor, School of Medicine, Hangzhou Normal University, Hangzhou, China; 2 Deputy director, Department of Primary Health Care, Health and Family Planning Commission of Zhejiang Province, Hangzhou, China

**Keywords:** China, internal market, pay for performance, primary health care, provider payment

## Abstract

**Aim:** To provide a framework for provider payment reform for primary care physicians in China. **Background:** Primary health care is central to health system reform and payment incentives have significant consequences for the equity and efficiency of it. **Methods:** This paper describes the special payments system for public primary health institutions and the subsequent internal salary remuneration to primary care physicians in China. Based on an analysis of the major challenges, we suggest a reform framework including the pattern of governance, and payments to primary health institutions and employed physicians. **Findings:** A mixed system of input-based and output-based payments to institutions would probably be appropriate under a long-term and relational contract with the government. It was also advised that internal remuneration is provided by a basic salary plus a bonus based on performance, and an extra-regional allowance. We hope that the results can be used to shift the passive budgeting of in-house staff within the public primary health institutions toward strategic purchasing.

## Introduction

Primary health care is central to China’s health system and has great potential to improve the well-being of the population (Zhang *et al*., 2017). Since the launch of a new round of health care reforms in 2009, China has made impressive progress in developing primary health care, including strengthening the infrastructure of primary health care facilities, and it is acknowledged that many accomplishments have already been achieved (Blumenthal and Hsiao, 2015). However, a report released by a joint team of five organizations, including the World Bank and World Health Organization, in 2015 recommended that one of the substantial challenges ahead was the transformation from a profit-driven, hospital-centered, and fragmented system to an efficient integrated primary care-based delivery system (World Bank Group *et al*., 2016). The literature suggests that this problem cannot be solved by simply shifting ownership to the private sector, or by simply encouraging providers – public and private – to compete with one another for individual patients (Eggleston *et al*., 2008). In contrast, payment incentives have significant consequences for the equity and efficiency of a health care system, and have recently been the focus of health policy reforms (Eggleston and Hsieh, 2004). However, the provider payment of primary care physicians (PCPs) has not been clearly clarified and studied to determine its particularity in China. For example, payments to PCPs are mediated indirectly by payments to the institution, with most PCPs being government employees. In 2015, community and township health centers received 5.59 and 10.63 million outpatient visits, respectively. Of these visits, 83.0 and 98.4%, respectively, were to government-run primary health institutions (PHIs) (National Health and Family Planning Commission of the People’s Republic of China, 2016). These PHIs are a kind of Public Service Unit (PSU), operating alongside the government (World Bank, 2005), and the provider payments to PCPs are to some extent made under a hierarchical ‘chain of command’ type of administration. However, there are deficiencies in the process, particularly due to the lack of appropriate incentives to improve efficiency and quality, and the absence of mechanisms to introduce incentives.

This paper describes the system of special payments to PHIs and the subsequent internal salary remunerations made to PCPs in China. Based on an analysis of the major challenges (accompanied by the zero-mark-up policy on drugs that was implemented in 2009), we suggest a mixed system of input-based (line-item budgets) and output-based payments (purchasing in internal or quasi-markets) to PHIs under a long-term and relational contract with the government. Through these mixed payments to PHIs, we propose that an internal remuneration is made to pay PCPs. Related data were obtained from the statistical yearbook of China (National Health and Family Planning Commission of the People’s Republic of China, 2016), and Health statistical data collections of Zhejiang Province (unpublished material), one of the 31 provincial regions of China, with a population of about 55 million. Our hope is that the results of this study will be used to restructure the provider payment system for primary health care and to develop a detailed plan of action to provide a more active and output/outcome-based payment system in China.

## Background

The provider payment system for primary health care in China can be divided into payments on an organizational level (PHIs) and internal salary remuneration on an individual level (PCPs). At the organizational level, public PHIs used to have three main sources of financing: direct government subsidies, service fees paid by users from out-of-pocket expenses and health insurance, and the mark-ups on drugs, which resulted in a fixed 15% or greater profit when prescribing and selling medicine (Yip *et al*., 2012). With the exception of Chinese herbal medicine, the income from drug mark-ups was lost after China’s new Essential Medicines Program was initiated in 2009, which had the aim of eliminating economic incentives to primary care providers by overprescribing drugs or prescribing unnecessary drugs. For example, drug expenses as a percentage of outpatient and inpatient expense dropped from 64.60 and 52.38% in 2009 to 57.88 and 44.83% in 2015, respectively (see Table 1). To compensate for the lost revenues as a result of the zero-mark-up policy, PHIs receive a government subsidy to support operational costs, including physical infrastructure, equipment procurement, human resource capacity, and public health services such as the National Essential Public Health Services Package (NEPHSP) (Yip *et al*., 2012; Barber *et al*., 2013). It should be noted that the gap between revenue and expenditure will only be subsidized by the government after assessing performance in the new provider payment system, but the corresponding revenue and expenditure needs to be determined before performance is assessed (Cheng *et al*., 2011; Mao and Chen, 2015). According to the statistical yearbook, financial subsidies from the government increased from 611.1 thousand RMB per PHI in 2009 to 3110.5 thousand RMB in 2015 (see Table 1). The financial subsidy as a percentage of total income increased from 19.30% in 2009 to 41.56% in 2015. If we exclude drug revenue from the total income, the financial subsidy as a percentage of total income was 59.70% in 2015(see Table 1).

**Table 1:** Income and expenditure of primary health institutions in China 2009–2015 (thousand RMB)

Items	2009	2010	2011	2012	2013	2014	2015
Number of PHIs (1)	42 046	43 289	43 617	43 869	43 974	44 060	44 110
Total revenue per PHI (2)	3166.7	3700.4	4429.3	5370.3	6066.2	6551.9	7483.8
Medical income (3)	2385.4	2586.2	2668.6	3127.1	3500.8	3793.4	4097.5
Drug income (4)	1449.2	1554.4	1465.4	1759.7	1946.6	2105.4	2273.2
Financial subsidy (5)	611.1	908.9	1530.9	2017.1	2322.4	2515.3	3110.5
As % of total income (6)=(5)/(2)×100%	19.30	24.56	34.56	37.56	38.28	38.39	41.56
As % of total income excluding drug income (7)=(5)/[(2)−(4)]×100%	35.58	42.35	51.65	55.87	56.37	56.57	59.70
Total expenditure per PHI (8)	3044.8	3576.8	4310.1	5159.9	5900.4	6316.6	7167.9
Drug expenses (9)	912.9	1061.9	1212.5	1594.0	1817.5	1951.0	2089.7
As % of outpatient expense (10)	64.60	63.00	56.83	58.38	57.98	57.90	57.88
As % of inpatient expense (11)	52.38	52.00	46.55	47.78	46.48	45.38	44.83
Net revenue rate from drug dispensing (%)(12)=[(4)–(9)]/(4)×100%	37.01	31.69	17.25	9.42	6.63	7.34	8.07
Net income per PHI (13)=(2)−(8)	121.9	123.6	119.2	210.4	165.8	235.3	315.9

PHIs=primary health institutions. Income and expenditure are not adjusted for inflation.

At the individual level, PCPs receive a salary that is controlled by two factors. The first is whether the PCP is a member of a PSU. Most PSUs in China have a special human resources management arrangement, referred to as the headcount quota system, which defines the total number of personnel approved by the government (World Bank, 2005). The headcount quota system is an important element in budgeting and defines funding allocations. For Zhejiang Province as an example, the total headcount quota increased from 33 304 in 2009 to 84 663 in 2015 (unpublished material). PCPs who hold a quota position benefit from a similar personnel system as civil servants in terms of recruitment, pensions, and remuneration (Weng, 2012). However, the welfare condition of contracted staff is not as good as those that hold quota positions.

Second, an employee’s salary will be decided by the amount of annual funding available per quota position and its composition (Yang and Dai, 2013; Chen *et al*., 2014). The wage is generally divided into two parts, Basic Performance Pay and Encouraging Performance Pay (Yang and Dai, 2013; Chen *et al*., 2014). The Basic Performance Pay is often fixed and generally accounts for 70% of the wage. However, it varies from person to person because it is linked to each individual PCP’s post, grade, professional title, and the local price index, which reflects the state of the local economy. Encouraging Performance Pay is flexible and logically depends on each PCP’s workload and performance, but that’s not often the case. A recent national survey showed the bonuses for PCP’s that constitute 30% (inter quartile range 20–50) of their income could have played a key part in incentivizing quality of care (Li *et al*., 2017). However, across institutions, these bonuses were most often determined by the quantity of care delivered rather than the quality (Li *et al*., 2017). Moreover, much performance management in local governments was process-oriented rather results-oriented, with supervision sometimes being weak and penalties for poor performance being exceptional (Yang and Dai, 2013; Zhao *et al*., 2013).

## Challenges

An analysis of China’s current primary care strategy indicates that, without significant reform, it is unlikely to lead to the development of an effective national program. Several critical problems have been identified, including payments at the organizational level (PHIs) and the subsequent internal salary remuneration at the individual level (PCPs).

The first challenge is fiscal sustainability. One of the typical features of the round of health care reforms after 2009 was an input-focused strategy, because, to some extent, China has concentrated on increasing public funding as a solution to cope with problems. Between 2009 and 2015, total health expenditure per capita and financial subsidy per PHI increased at an annual rate of 14.4% (from 1314.3 to 2951.8 RMB) and 31.1% (from 611.1 to 3110.5 thousand RMB), respectively. Both of these increases are larger than the annual rate of gross domestic product per capita (11.3%, from 26 222 RMB in 2009 to 49 992 RMB in 2015). Spending more money may appear easier in the context of surging government revenues, and such a policy has contributed to many successes, including improved equity and accessibility to health care. However, China is now facing greater challenges, with the high growth rates in health expenditure in recent years being difficult to sustain due to an economic slow-down (Yip *et al*., 2010). The largest challenges are the fiscal sustainability and increasing weaknesses in health delivery systems, including allocative inefficiency, failure to reach groups living in poverty, and poor responsiveness.

The second challenge is the poor performance in distributing health resources. Between 2009 and 2015, outpatient visits per capita increased by 33.3% (from 4.2 visits to 5.6 visits per year). However, the percentage of outpatient visits delivered in hospitals among all health care facilities increased from 35.02 to 40.08% from 2009 to 2015, while the proportion accounted for by PHIs dropped from 61.82 to 56.44% (see Table 2). This is far from the 80% recommended by the World Health Organization (2008). More importantly, PHIs are becoming marginalized, with hospitals taking a principal role in providing inpatient services. Population-based hospitalization rates in China rose 54.5% (from 9.9 to 15.3%) between 2009 and 2015, but the percentage of services accounted for by hospitals among all health care facilities increased from 64.03 to 76.41%, while the proportion of services accessed in PHIs dropped from 31.01 to 19.17% (see Table 2).

**Table 2:** Number of outpatient visits and inpatients in health care facilities (million)

Items	2009	2010	2011	2012	2013	2014	2015
Total outpatient visits	5487.67	5837.62	6271.23	6888.33	7314.01	7601.87	7693.43
#Hospitals	1921.94	2039.63	2258.84	2541.62	2741.78	2972.07	3083.64
As % of total	35.02	34.94	36.02	36.90	37.49	39.10	40.08
#PHIs	3392.37	3611.56	3805.60	4109.21	4324.31	4363.95	4341.93
As % of total	61.82	61.87	60.68	59.65	59.12	57.41	56.44
#Others	173.37	186.43	206.79	237.51	247.92	265.85	267.86
As % of total	3.16	3.19	3.30	3.45	3.39	3.50	3.48
Total inpatients	13.26	14.17	15.30	17.81	19.22	20.44	21.05
#Hospitals	8.49	9.52	10.76	12.73	14.01	15.38	16.09
As % of total	64.03	67.19	70.30	71.45	72.90	75.22	76.41
#PHIs	4.11	3.95	3.78	4.21	4.30	4.09	4.04
As % of total	31.01	27.87	24.68	23.63	22.38	20.03	19.17
#Others	0.66	0.70	0.77	0.88	0.91	0.97	0.93
As % of total	4.96	4.94	5.02	4.92	4.72	4.76	4.42

PHIs=primary health institutions.

The third challenge is related to the ‘soft’ budgets and low residual claimant power. The term ‘soft’ refers to the lack of enforced financial responsibility (Eggleston *et al*., 2009). For the provider payment system at the organizational level, public PHIs are subsidized by line-item budgets, such as physical infrastructure, equipment procurement, human resource capacity, and the gap between revenue and expenditure. To manage the risk of ‘soft’ budgets, it is often regulated that revenue and expenditure must be predefined (Chen *et al*., 2014). In reality, it is impossible to predict these values accurately due to the uncertainty of the exact health services that will be required and their corresponding income and expenditures. Payments to PHIs are therefore often based on previous budgets. PHIs also have a low residual claimant power, that is, the ability to both retain savings and take responsibility for the debt. If PHIs generate extra revenue by making savings, line-item budgets for the gap between revenue and expenditure will be reduced. In such cases, they will be subsidized less the next year because it is deemed that these PHIs have the capability for self-improvement and the funding is not needed. Such conditions are not conducive to generate savings and efficiency because they result in perverse ‘Ratchet effects’ (staff holding back performance) (Meyer and Vickers, 1997).

The fourth challenge regards the provider payment system of the NEPHSP. The NEPHSP is budgeted per capita (resident population) after 2009, and it aims to deliver essential public health programs to all citizens regardless of their geographic location, gender, earnings, etc. In theory, PCPs are paid by the quantity and quality of the services they provide, but in practice, the payment is not effectively related to the services provided because PCPs are mainly paid a salary, which is related to the quota system. Such a system inevitable influences the quality and effectiveness of the services.

The fifth challenge is the low incentive to PCPs provided by the Pay for Performance system. Pay for Performance is not seen as a strong incentive for several reasons, such as the rigidity of salary payments, low levels of management autonomy in deciding the level of salary and its composition, and income disparities between those with and without quota positions (Yang and Dai, 2013). A quota position serves as the basis for budgeting, recruitment, pensions, and remuneration. In reality, there are many contracted staffs without a quota position and their pay is not as good as those with a quota position. For Zhejiang Province as an example, 29.4% (25,210 of 85,700) of the staff do not have quota positions and their remuneration levels were only about 60% of those staff with quota positions in 2015 (unpublished material). Low job satisfaction and high occupational burnout are also widespread. A systematic review of 13 studies showed a decline in job satisfaction among urban PCPs after the 2009 health care reforms (Zhang *et al*., 2016). Another study noted that, of 10 626 urban and rural PCPs, 4307 (41%) felt highly exhausted, 3974 (37%) felt highly depersonalized, and 3616 (34%) felt that they highly lacked personal accomplishment.

## Reform framework

The provider payment system should be reformed, with a design according to the delivery units of the service, such as districts, PHIs, and PCPs. Property rights, governance structure, market environment, and funding arrangements are often connected with provider payments (Preker *et al*., 2000; Hammer and Jack, 2001; Jegers *et al*., 2002; Robyn *et al*., 2016). The proposed framework should be reviewed to determine its suitability to the surrounding area. Preker and Harding proposed several innovations in health service delivery, including autonomization, corporatization, and privatization (Preker and Harding, 2003). Corporatization and privatization is a radical approach in the short term because the governance capacity and its accountability are not matched for the PHIs in China. Autonomization is probably a more suitable idea in terms of a progressive reform strategy. In theory, health care providers will make allocative decisions more efficiently if they are awarded more managerial freedom, and they will work harder if their remunerations are tightly linked to measured outputs. This study developed a framework for provider payment reform for PCPs in China, including the pattern of governance and payments to PHIs and PCPs.

### Pattern of governance

**Figure 1:**
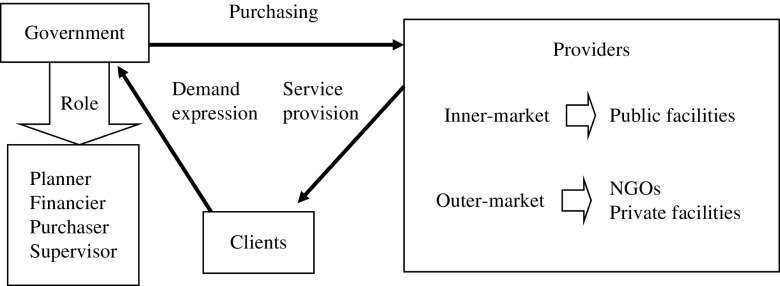
The pattern of governance for health facilities. NGO=non-governmental organizations.

In provider–payer reforms worldwide, financial and performance incentives have been used on both the supply and demand sides (Meyer, 1993; De *et al*., 2006; Takian *et al*., 2015). Government is expected to have a central role in developing organized modes of health care financing and provider payment systems. We propose a governance pattern for the three parties, that is, governments, PHIs, and clients (See Figure 1).

Once the functions of financing and provision are separated, the provider can achieve greater management autonomy, decision rights, and residual claims, accompanied by accountability and the stress of being in a competitive market. In these circumstances, a form of contract or agreement is essential to bind the purchaser and provider. The contract may help to drive performance by allowing priorities to be identified and targeted, offering greater accountability to the government and public, focusing attention on outcomes and quality, establishing a systematic approach to monitor performance, and creating autonomy in encouraging responsible staff initiatives and motivation (World Bank, 2005; Weng, 2012). However, it is also easy for transaction costs to escalate. Transaction cost is the key theoretical element in New Institutional Economics, originated by Coase and developed by Williamson (Richter, 2005). The net effect of the total transaction costs, including external and internal costs, has been applied to compare the different contractual arrangements in a number of health care contexts (Dollery, 2001; Stiles *et al*., 2001; Donato, 2010). External costs include those arising from incomplete contracts, the complexity of the diversified services, small numbers bargaining, asset specificity, the frequency of exchanges, and the underlying information asymmetries. Internal costs include those of human resource activities, such as hiring and staffing, training, evaluating, and administering programs. An internal market with flexible, longer-term, and relational contracts are probably more operable and reliable in China than contracting-out to private facilities and non-governmental organizations (NGOs) (Ashton, 1998; Porter *et al*., 2013). The reasons for this may include the majority of PHIs being public facilities, the capacity of the governance and administrative systems being unsuitable in the initial stages, and the relatively low transaction costs for frequent and repeated exchanges. Governments and PHIs in the internal market are less likely to risk undermining a potentially long-term relationship with opportunistic behavior. A good mechanism for internal markets and optimal governance structure will facilitate the transition from the contracting-in of government-run PHIs to the contracting-out to private facilities and NGOs.

### Payment to PHIs at the organizational level

**Figure 2:**
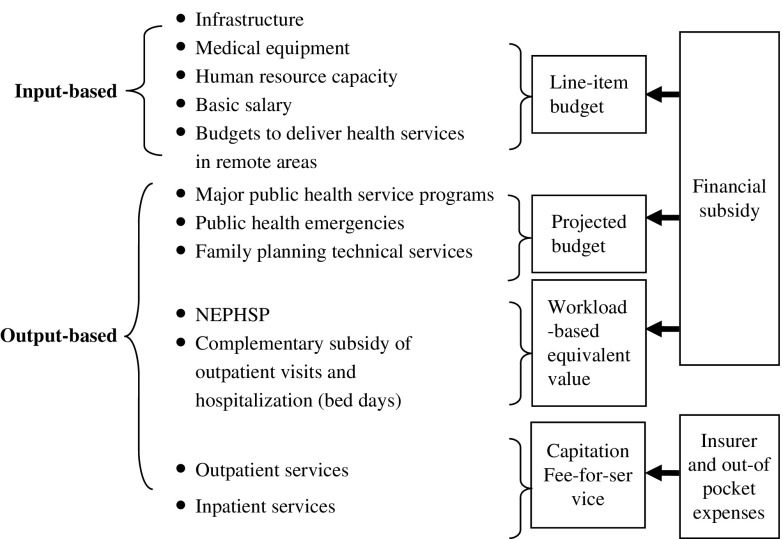
Payments to primary health institutions. NEPHSP=National Essential Public Health Services Package.

Many studies have attempted to identify optimal payment methods, with both efficiency and quality being an objective function of the payer (Gosden *et al*., 1999; Gosden *et al*., 2001; Eggleston and Hsieh, 2004; Dan and Savi, 2013). The general conclusion is that mixed provider payments are necessary to optimally balance cost and quality (Eggleston and Hsieh, 2004). However, there is no easy solution in a transitional context. It requires a good understanding of the nature of the work and careful consideration of the advantages and inherent problems of various payment methods. Moreover, the theory of path-dependency shows that the set of decisions for any given circumstance is limited in varying degrees by the decisions made in the past (Cacace and Frisina, 2010). In China, most PHIs are public and the payments create incentives to increase the inputs, such as infrastructure and human resources (Li *et al*., 2017). Based on some local experiences (Yin *et al*., 2015; Wang *et al*., 2016; Jiang *et al*., 2017; Zhang *et al*., 2017) and a pilot study in Zhejiang Province (unpublished material), we suggest a mixed system of input- and output-based provider payments (see Figure 2), simply because output-based payments can counter the adverse incentives of input-based payments, while retaining their desirable features.

Input-based provider payments are essential to the accessibility and equity of primary health care in China. China once had a strong primary health system that was a model for other nations, but there was a shift from rural to urban facilities and from primary care-based services to public hospital-centered care after 1978 (Liu, 2004; Eggleston *et al*., 2008; Blumenthal and Hsiao, 2015; World Bank Group *et al*., 2016). Effective primary health care is very important to cope with a series of new and serious challenges, including industrialization, urbanization, an aging population, and changes in the spectrum of diseases. For input-based provider payments, we propose line-item budgets. Line-item budgets are characterized by the allocation of resources to PHIs to provide the items (Waters *et al*., 2004). Based on the health care reforms made in 2009 and previous provider payment systems (Eggleston *et al*., 2008; Yip *et al*., 2010; Blumenthal and Hsiao, 2015; World Bank Group *et al*., 2016), the items we propose include infrastructure, medical equipment, human resource capacity, basic wages, and special budgets to deliver health services in remote areas. All these items should be financed through a basic grant according to local conditions. The benefits of line-item budgets are the increasing accessibility and equality of health services, and a reduction in the cost of choosing appropriate providers (Waters *et al*., 2004). Basic wages are a specialized line-item budget in the input-based provider payment system and will guarantee a basic income for PCPs. All of these advantages are potentially important, but also create problems, such as low responsiveness to the needs of patients and payers, and a lack of flexibility in resource use. An output-based provider payment system was designed to counter these adverse features.

An output-based provider payment system should be designed according to the services provided. In Figure 2, output-based provider payments can be differentiated into two categories based on sources of funding: finance-funded services and insurance-provided services. Major public health service programs, public health emergencies, and family planning technical services are all funded by government and are managed as projects. We proposed that these services should be budgeted as projects beforehand or the costs settled after delivery. For the NEPHSP, we propose a provider payment referred to as the workload-based equivalent value, based on methods used by Yin (Yin *et al*., 2015). The actual amount paid is a function of the number of workload-based equivalent values (total points) provided, with a value established for a point and a comprehensive quality evaluation index provided for services. Five steps were needed to calculate the value of a point based on historical data (usually the past one to three years). (1) Determine the standard service protocols of all NEPHSP services. (2) Define the workload indicators needed for a set of standard activities for these services (Yin *et al*., 2015), and their equivalent value compared with a standard clinic visit (one point). (3) Calculate the points of every NEPHSP service based on historical data, which is then multiplied by the amount of services provided and their corresponding equivalent value. (4) Calculate the total points by summing the points of every NEPHSP service. (5) Divide the total number of points by the revenue allocated to the NEPHSP services to produce the value for one point.

We also proposed an output-based payment to pay for medical services including drug prescriptions. The drug revenues lost following the imposition of the zero-mark-up policy have largely been subsidized by the government in terms of filling the gap between revenue and expenditure after 2009. This is very different to the reimbursement made for the zero-mark-up policy in county-level hospitals, where the lost drug revenue has mainly been reimbursed by increasing the prices of medical services (Li *et al*., 2015). To create an output-based payment as a subsidy for the zero-mark-up on drugs, a workload-based equivalent value was introduced to reimburse PHIs based on the number of outpatient visits and hospitalizations (bed days) they provided. We also considered a standard clinic visit as one point. The equivalent value for a hospitalization bed day was defined by comparing its workload indicators (Yin *et al*., 2015) with a standard clinic visit. The actual amount paid was also a function of the total number of points for health services and the value established for one point.

Clearly, as medical insurance coverage widens and the funds devoted to insurance increase, the ability to use provider payments for medical services will also increase. Fee-for-service (FFS) is the dominant payment method for medical services, including drug prescriptions. Most western countries employ a combination of FFS, fixed salary, and per capita subsidies to finance the services of general practitioners (Gosden *et al*., 1999). The context of provider payments is different because most PCPs are remunerated from PHIs in the form of a salary. We propose a mixture of FFS and per capita (such as resident population or effective contracted population at the early stage of general practitioner system) payment rather than a pure FFS system to remunerate medical services at the PHI level. FFS financing ensures that PCPs have an incentive to offer services, especially for profitable activities such as prescribing diagnostic tests and drugs (Gosden *et al*., 2001). Per capita payment commits PCPs to make services available for patients, by making them responsible for patients. The outcome of a mixed FFS and capitation system may be somewhere between over- and under-treatment.

### Payment to PCPs at the individual level

Most PCPs are employed by PHIs and salary-based systems are the main provider payment. In western countries, such systems are commonly thought to provide few incentives to encourage the delivery of services as salaries commonly depend on the qualification and task profile of the physician and not on performance (Gosden *et al*., 1999). However, salary payment is likely to be administratively simpler than other provider payments such as an FFS system (Gosden *et al*., 1999). Moreover, it appears to be very well suited in the institutional context of most PHIs being public facilities in China and the path-dependence of the original payments. In this study, we attempted to optimize the salary payment process to maximize the advantages and reduce the inherent problems.

First, some inherent problems associated with salary payment, such as the lack of incentive for improving performance can be alleviated to some extent by output-based provider payments at the facility level. These output-based payments include projected-budgeting, workload-based equivalent values, capitation, and FFS that pertain to the different services provided. The inherent problems of salary payment can be adjusted by organizational management and control. For example, PHI managers will have more management autonomy, decision rights, and residual claims after the reform.

Second, it is advised that salary payments should consist of a basic salary plus a bonus based on performance. The total salary and its performance-based proportion should be suited to local conditions and managed locally. Salary payments to PCPs should interact with the mixed payments at the facility level. To include some social security benefits in the basic salary, we propose an input-based payment from subsidies made at the facility level. In view of the differences in PCPs, the basic salary should be determined locally and individually, which would typically depend on the qualification and task profile of the PCPs. For the performance bonus, we propose a mixed payment system comprising a workload-based equivalent value and additional remuneration. These payments would be introduced to remunerate PCPs for medical services and other projected-budgeted costs. The performance pay for PCPs is multiplied by the total workload-based equivalent values (total points) provided, the value established for a point and a comprehensive quality evaluation index for every PCP. The use of an index would enable an evaluation by the PHI manager based on performance. Additional remunerations are proposed to pay for special services and additional tasks.

Third, we suggest an extra allowance system at the regional level to pay special groups an additional amount above their regular salary payments and so ensure efficiency and equity. This extra allowance system should cover directors of PHIs, outstanding employees, and health professionals in remote areas. As PHI directors will get more management autonomy and decision rights, a management bonus or penalty could be implemented in accordance with their performance. Rural and remote areas are disadvantaged by their inability to attract and retain PCPs. We therefore propose an extra payment is made in such areas as an incentive to recruit, train, and retain PCPs in such areas. For example, the government offers to reimburse tuition fees to ensure that new medical students are enrolled to become doctors, nurses, and health practitioners in medically underserved areas. We also suggest a retention payment, taking into account both the length of service and the remoteness of the area the PCP works in.

This paper has certain limitations. In the light of methodological concerns about the reform framework that has been studied, it is still necessary to carefully evaluate future models and real-life experience. For instance, many technical and political factors might affect the transformation from passive budgeting to strategic purchasing within the public PHIs. These factors may include risk adjustment; the balance of input and output-based payments; participation of physicians and patients in decision making; institutional challenges (such as the slow reform process in PSUs); supervision and quality assurance systems. However, this paper is not intended to be a comprehensive program that specifies the future change, but rather a reform framework toward improvement.

## Summary

We have provided a framework for provider payment reform for PCPs in China, including the pattern of governance, and payments to PHIs and PCPs. For the pattern of governance, financing and service provision are proposed to be separated through an internal market with flexible, longer-term and relational contracts between government and PHIs. Payments should be made to PHIs at the organizational level, including a mixed system of input-based (line-item budgets) and categorized output-based payments. The NEPHSP is advised to make payments by a workload-based equivalent value from government subsidies. A mixture of FFS and per capita payments instead of a pure FFS system is proposed to remunerate medical services at the PHIs level. Salary-based payments appear to be suitable in China, but should be optimized by a basic salary, plus a bonus based on performance and an extra-regional allowance for designated groups such as directors of PHIs, outstanding employees, and health professionals in hard to reach or remote areas.

## Conflicts of Interest

None.

## Financial Support

This work was supported by grants from the National Natural Science Foundation of China (No. 81301466).
